# Absence of chloride intracellular channel 4 (CLIC4) predisposes to acute kidney injury but has minimal impact on recovery

**DOI:** 10.1186/1471-2369-15-54

**Published:** 2014-04-03

**Authors:** John C Edwards, Jonathan Bruno, Phillip Key, Yao-Wen Cheng

**Affiliations:** 1 Kidney Center and the Department of Internal Medicine, University of North Carolina, Chapel Hill NC, USA; 2 Division of Nephrology, Department of Internal Medicine, Saint Louis University, St. Louis, MO, USA

**Keywords:** CLIC4, Acute kidney injury, Glomerular endowment, Peritubular capillary network, Transforming growth factor β

## Abstract

**Background:**

CLIC4, a member of the CLIC family of proteins, was recently demonstrated to translocate to the nucleus in differentiating keratinocytes where it potentiates TGFβ-driven gene regulation. Since TGFβ signaling is known to play important roles in the fibrotic response to acute kidney injury, and since CLIC4 is abundantly expressed in kidney, we hypothesized that CLIC4 may play a role in the response to acute kidney injury.

**Methods:**

Previously described *Clic4* null mice were analyzed for the effect of absence of CLIC4 on growth, development and response to kidney injury. Kidney size, glomerular counts and density of peritubular capillaries of matched WT and *Clic4* null mice were determined. Cohorts of WT and *Clic4* null mice were subjected to the folic acid model of acute kidney injury. Extent of acute injury and long term functional recovery were assessed by plasma blood urea nitrogen (BUN); long term fibrosis/scarring was determined by histochemical assessment of kidney sections and by residual renal mass. Activation of the TGFβ signaling pathway was assessed by semi-quantitative western blots of phosphorylated SMADs 2 and 3.

**Results:**

CLIC4 is abundantly expressed in the apical pole of renal proximal tubule cells, and in endothelial cells of glomerular and peritubular capillaries. CLIC4 null mice are small, have smaller kidneys with fewer glomeruli and less dense peritubular capillary networks, and have increased proteinuria. The *Clic4* null mice show increased susceptibility to folic acid-induced acute kidney injury but no difference in recovery from acute injury, no nuclear redistribution of CLIC4 following injury, and no significant difference in activation of the TGFβ-signaling pathway as reflected in the level of phosphorylation of SMADs 2 and 3.

**Conclusions:**

Absence of CLIC4 results in morphologic changes consistent with its known role in angiogenesis. These changes may be at least partially responsible for the increased susceptibility to acute kidney injury. However, the absence of CLIC4 has no significant impact on the extent of functional recovery or fibrosis following acute injury, indicating that CLIC4 does not play a major non-redundant role in the TGFβ signaling involved in response to acute kidney injury.

## Background

Genetically defined mice are powerful tools that are capable of identifying roles of specific proteins in physiologic processes. However, interrelations between genes and complex phenotypes can be indirect, multifaceted, and challenging to unravel. One such complex phenotype is the susceptibility to and recovery from acute kidney injury (reviewed in [1-4]). Toxic or ischemic injury to kidney tubules triggers a cascade of events which include apoptosis and sloughing of injured cells, “dedifferentiation” of surviving cells which then proliferate and migrate to repopulate the tubule, and finally re-differentiation [1]. This process involves mediators generated by both endogenous kidney cells and by infiltrating white blood cells which are instrumental in both the initial injury and the long term recovery [1-4]. Recovery may be incomplete and accompanied by significant interstitial fibrosis and scarring that leads to chronic kidney disease and increased susceptibility to future renal insults [2]. Injury and recovery is not limited to the epithelial cells, but also involves the endothelial cells of the peritubular capillaries. Failure of recovery of this compartment results in rarefication of the peritubular capillary network and is associated with poor functional renal recovery [5,6]. Identification of genes and proteins involved in both susceptibility to acute injury and subsequent chronic kidney scarring may lead to insights into treatment and/or prevention of these important human disease states.

CLIC4 is a member of the CLIC family of proteins which were originally identified as chloride channels of intracellular membranes [7,8]. Over the years, a variety of diverse yet not entirely mutually consistent data have been presented about CLIC4. Thus CLIC4 has been reported to be in endoplasmic reticulum, trans-Golgi network, caveolae, mitochondria, dense-core secretory vesicles in the central nervous system, and nuclei of differentiating keratinocytes [9-13]. Purified CLIC4 has been reported to function as a channel *in vivo* but details of channel properties are not consistent among the reports [14,15]. It has variously been proposed to function as a channel of intracellular membranes [16,17], as a regulator of apoptosis [18-20], as a cytoskeletal component [21,22], and as a modulator of gene expression during differentiation of myofibroblasts [23]. Although the function of CLIC4 is still uncertain, it has been most convincingly implicated in two distinct cellular processes: the intracellular membrane trafficking leading to tubulogenesis of endothelial cells [17,24,25], and potentiation of transforming growth factor β (TGFβ) signaling during keratinocyte differentiation and wound healing in the skin [23,26,27].

Angiogenesis and TGFβ signaling are both known to be relevant to acute kidney injury. Angiogenesis is critical to development of the kidney, particularly in formation of glomeruli, and glomerular endowment is known to affect susceptibility to acute kidney injury (AKI); peritubular capillary injury is an important component of the initial injury and angiogenesis of this compartment in response to acute injury may aid in recovery [6,28]. TGFβ signaling has long been recognized as an important component in the response to acute kidney injury, playing a role in driving the fibrosis and scarring following injury [29-31]. Based on these observations, our central hypothesis is that CLIC4 is important to the susceptibility and response to kidney injury.

We have previously reported the generation of mice in which the gene for CLIC4 has been disrupted [17]. We chose to use our *Clic4* null mice to investigate the role of CLIC4 in the kidney. In the results presented here, we find that CLIC4 is expressed in proximal tubule cells as well as endothelial cells of both peritubular and glomerular capillaries. *Clic4* null mice have smaller kidneys with fewer glomeruli and less dense peritubular capillary network, consistent with a role for CLIC4 in angiogenesis during development of the kidney. The *Clic4* null mice were found to have albuminuria but do not have prominent glomerular ultra-structural abnormalities that are often seen in proteinuric states. *Clic4* null mice show increased susceptibility to folic acid-induced acute kidney injury. However we did not find compelling evidence for a role for CLIC4 in either the functional recovery or the fibrosis and scarring following injury, indicating that CLIC4 does not play a critical non-redundant role in the TGFβ signaling that drives scarring following injury.

## Methods

### Mice

Generation of the mice carrying a disrupted *Clic4* gene has been previously described [17]. Male and female *Clic4*^*(-/-)*^ mice in the CD1 background were crossed with CD1 WT mice (Charles River) to generate newly outbred *Clic4*^*(+/-)*^ mice. Multiple pairs of non-sibling newly outbred *Clic4*^*(+/-)*^ mice were mated and *Clic4*^*(-/-)*^ and *Clic4*^*(+/+)*^ mice selected from this F1 generation. Non-sibling F1 *Clic4*^*(+/+)*^ or *Clic4*^*(-/-)*^ mice were mated to generate the F2 *Clic4*^*(+/+)*^ (wild type, WT) and *Clic4*^*(-/-)*^ (*Clic4* null) mice that were used in all these experiments. Animals to be studied were randomly chosen from the available population. The *Clic4* genotype of each mouse was confirmed by polymerase chain reaction at the end of each experiment using DNA prepared from tail snips as previously described[17]. Mice were maintained in conventional static microisolator cages (1–5 mice per cage) with cob bedding and a paper supplement for enrichment and nesting, light/dark cycles of 12 hours, temperate regulated at 70 F, with continuous access to water and standard mouse chow. Animal health was actively monitored by husbandry and veterinary staff. The animal facilities are registered with the USDA, follow the regulations set out in the US Government Principles, the Guide for Care and Use of Laboratory Animals and the US Public Health Service Policy as required by National Institutes of Health and the Office of Laboratory Animal Welfare, and are fully accredited by the Association for Assessment and Accreditation of Laboratory Animal Care International. All mouse studies were in compliance with protocols approved by the Institutional Animal Care and Use Committees of the University of North Carolina at Chapel Hill and/or St. Louis University, as appropriate.

### Antibodies and lectins

AP255 and AP1089, the affinity-purified rabbit polyclonal antibodies to CLIC4 and CLIC1, respectively, have been previously described [17,32]. Commercial antibodies were as follows: Goat polyclonal antibody to mouse albumin, Bethyl Labs (Montgomery TX) #A90-134; rat monoclonal antibody to CD31 clone MEC13.3, Pharmingen (San Jose, CA) #550274; mouse monoclonal antibody to PCNA, Cell Signaling Technology (Danvers, MA) #2586; rabbit monoclonal antibody to Smad2/3, Cell Signaling Technology #8685; rabbit monoclonal antibody to phospho-Smad2/3, Sigma-Aldrich #SAB4504208; mouse monoclonal antibody to GAPDH, Santa Cruz Biotechnology (Santa Cruz, CA) #SC-32233; goat polyclonal antibody to CLIC5, Santa Cruz Biotechnology #SC-65041 Alexa Fluor 488 anti rat IgG, Life Technologies (Grand Island, NY); Cy5 goat anti rabbit IgG, Jackson Labs (Bar Harbor, ME) #111-175-144; FITC-lectin from Lotus tetragonolobus (LTA), Vector Labs (Burlingame CA) #FL-1321; Alexa Fluor 594-isolectin B4 from Griffonia simpicifolia (IB4), Life Technologies, #I21413; HRP-conjugated secondary antibodies, Thermo Scientific Pierce.

### Immunolocalization

Adult mice were anticoagulated with 500 units of heparin by intraperitoneal injection 10 minutes prior to euthanasia by CO_2_ inhalation. The mice were perfused with 30–50 ml of phosphate buffered saline through the left ventricle via a short-bevel 22 Ga. needle and exiting the lacerated right atrium. The mice were then perfused with 30 ml of fresh PLP fixative (2% paraformaldehyde, 75 mM sodium phosphate, 75 mM lysine, 10 mM sodium periodate, pH 7.4). The kidneys were removed, divided into 3–4 pieces, and further fixed in PLP for 24 hours. For extended storage, the fixative was replaced with 0.05% paraformaldehyde in PBS to minimize antigen masking.

Sections 100 μm thick were made using a Leica VT 1200 vibratome. Sections were washed in PBS and blocked and permeabilized for 2 hours in an excess volume of Superblock (Thermo Scientific Pierce #37535) supplemented with 0.5% (v/v) Triton X-100. All subsequent antibody incubations and washes were performed in 15–50 μl drops on hydrophobically-masked slides in TNTFB2^+^ (50 mM tris[hydroxymethyl]aminomethane, 200 mM NaCl, 0.1% coldwater fish skin gelatin, 1% bovine serum albumin, 0.05% Tween-20, 0.5 mM MgCl_2_, 0.5 mM MnCl_2_, 0.5 mM CaCl_2_, pH 7.5). Incubation with the primary AP255 antibodies was done overnight at 4°C; samples were washed six times over more than one hour; incubation with the secondary antibody and lectins was for two hours at room temperature (cy5-conjugated goat anti rabbit, 20 ug/ml; FITC conjugated LTA, 40 ug/ml; and Alexa Fluor 594-conjugated IB4, 40 ug/ml) followed by washing as before. Because of LTA incompatibility with glycerol mounting compounds, final mounts were made in 100 mM tris[hydroxymethyl]aminomethane, 100 mM NaCl, 10 mM ascorbic acid, 0.5 mM MgCl_2_, 0.5 mM MnCl_2_, 0.5 mM CaCl_2_,and 500 ng/ml 4′,6-diamidino-2-phenylindole (DAPI) pH 7.5. Number 1.5 coverslips were sealed using quick-setting epoxy and imaged using an Olympus Fluoview 1000 scanning confocal microscope in the Research Microscopy Core at St. Louis University School of Medicine.

### Glomerular counts

Glomeruli were labeled with Alcian Blue using a variant of established methods [33,34]. The formulation of the dye known as Alcian Blue has changed since its original manufacture [35]. Unlike the original, the currently available compound is not soluble in saline. Alcian Blue 8GS (Applichem, Darmstadt Germany) was dissolved at 5 mg/ml in 5% dextrose, centrifuged at 12,000 g for 10 minutes, and then passed through a 0.22 micron filter. Mice were given a subcutaneous injection of 150 units of heparin 10 minutes prior to euthanization by CO_2_ inhalation. Mice were perfused through the left ventrical with 10 ml of PBS, followed by 10 ml of 5% dextrose, 4 ml of the Alcian Blue solution, and finally 15 more ml of 5% dextrose. The kidneys were excised and, after removal of the capsule, cut in half, soaked in 5 ml of 1% ammonium hydroxide for 2 hours, then transferred to 5 ml of 6 N HCl and incubated at 37C for 1.5 hours. The resulting suspension was vigorously vortexed to break up clumps, 25 ml of water was added and the suspension was placed at 4°C overnight. 200 μl aliquots of the macerated kidney suspension were placed on a grid and glomeruli in each aliquot counted under a low power objective. Glomeruli were readily distinguished by their blue staining. Additional aliquots were assessed until a minimum of 500 glomeruli from each pair of kidneys were counted. The person counting was blinded to the genotypes of the samples.

### Peritubular capillary density

Frozen unfixed longitudinal sections through the center of the kidney were prepared from a set of age- and sex- matched WT and *Clic4* null mice. Sections were fixed on the slide with 100% methanol at -20°C for 5 minutes. The sections were probed with rat monoclonal antibody to CD31, followed by an Alexa Fluor488-conjugated anti-rat IgG antibody. A set of contiguous images were obtained which spanned the length of the kidney. Images from all mice were collected and processed identically. ImageJ software (http://imagej.nih.gov/ij/) was used to determine the fractional surface area of each section that stained for CD31, after excluding glomeruli, large vessels, artifacts, and edges. The person carrying out the image analysis was blinded to the genotypes of each set of sections.

### Induction of acute kidney injury

Age- and sex- matched 6 to 12 week old mice were subjected to folic acid-induced acute kidney injury using an established protocol [36-38]. Within a few days before the induction of injury, a blood sample was obtained for initial blood urea nitrogen (BUN) determination. Unless otherwise noted, a sterile solution of 30 mg/ml folic acid (Sigma Aldrich, St. Louis MO Cat # F8758) in 300 mM bicarbonate was administered as a single intraperitoneal injection at a dose of 250 mg per kg body weight. Treated mice were maintained in group cages with ad libitum access to water and standard mouse chow. Blood samples were obtained by tail vein nicking at 2, 7, and 21 days after the folic acid injection.

### Measurement of renal fibrosis following injury

Mice were euthanized on day 21 following folic acid treatment. Formalin fixed, paraffin embedded midline longitudinal kidney sections were stained with Mason’s trichrome. Standard light micrographs were collected with a 4× objective. A composite image covering the entire section was generated from individual images. ImageJ software was used to determine the fractional area of each section that stained blue.

### Semi-quantitative western blotting

Kidney homogenates were generated by grinding freshly isolated kidney in RIPA buffer (1/2 kidney in 0.5 ml) supplemented with Mammalian Proteinease Inhibitor Cocktail (Sigma-Aldrich #P8340) and Phosphatase Inhibitor Cocktail 3 (Sigma-Aldrich #P0044). Insoluble material was removed by centrifugation. Total protein concentration was determined using BCA reagent (Thermo Scientific Pierce, Rockford, IL). Fifty micrograms of each sample were separated on 10% SDS-PAGE gels, blotted to PVDF membrane, blocked with 5% nonfat dried milk in TNT (200 mM NaCl, 50 mM Tris[hydroxymethyl]aminomethane, 0.1% Tween 20 pH 7.5) and probed sequentially with antibodies to CLIC4, CLIC1, CLIC5, SMAD2/3, phosphorylated SMAD2/3, PCNA, and GAPDH. Proteins were detected with HRP-conjugated secondary antibody and Supersignal Extended West Dura chemiluminescent reagent (Thermo Scientific Pierce), using a Protein Simple digital luminescence imager. Membranes were stripped between each iteration of detection by incubation in 2% SDS, 50 mM Glycine pH 2.5 for 30 minutes at room temperature, followed by washing with TNT and re-blocking with 5% nonfat dry milk in TNT. The intensity of luminescence signals were normalized to the GAPDH signal in the same lane.

### Clinical lab values

Blood and urine clinical lab testing including BUN, plasma albumin, urine total protein, and urine creatinine was performed using the services of the animal core lab at the University of North Carolina. Blood and urine β2 microglobulin was determined using an ELISA-based kit from Uscn Life Sciences (Houston, TX, cat. # E90260Mu). Urine albumin concentration was determined by quantitative western blotting as follows. Urine containing 1.5 μg of creatinine from each mouse was separated by SDS-PAGE and blotted to nitrocellulose. A series of standards diluted from mouse plasma with known albumin concentration was run on the same gel. The blot was probed with an antibody to mouse albumin, developed with HRP-conjugated secondary antibody, and quantitatively detected by chemiluminescence using a Protein Simple digital imager.

### Statistics

Unless otherwise noted, values are reported as means ± the standard error of the mean. Wilcoxon rank sum test was used to determine the significance of the difference in the 48 hour BUN values and 21 day fractional scarring between WT and *Clic4* null mice since these data are clearly not normally distributed. P values for differences among proportions were determined using two-tailed Fisher’s exact test. Differences of intensity of western blot signals were analyzed using Analysis of Variance methods since these data contained multiple equivalent groups. All other comparisons were analyzed with two tailed, unpaired Student’s T-test. All statistical methods were as described by Armitage [39].

## Results

### Distribution of CLIC4 in normal mouse kidney

**Figure 1:**
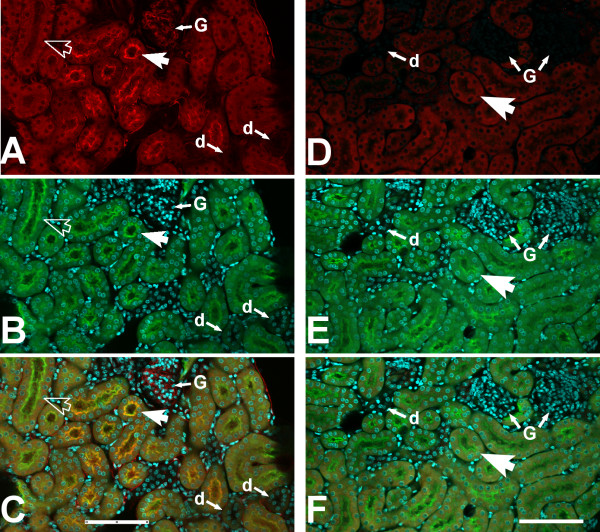
**Immunolocalization of CLIC4 in mouse kidney, low power images.** Vibratome sections of mouse kidney stained with antibody to CLIC4 (red) plus markers for the proximal tubule brush border (LTA, green), and nuclei (DAPI, cyan) were observed with a 20× objective under confocal microscopy. The panels on the left **(A-C)** are from a WT mouse, the panels on the right **(D-F)** are from a *Clic4* null mouse. **A** and **D**: CLIC4 signal. **B** and **E**: Merged LTA (green) and DAPI (cyan) signals. **C** and **F**: Merged image with all signals. The large arrows indicate proximal tubules, identified by brush border staining with LTA. In panels **A**-**C**, the solid large arrow indicates a LTA-positive proximal tubule that shows apical staining for CLIC4; the open large arrow indicates a LTA-positive proximal tubule that does not show apical CLIC4 staining. Glomeruli are designated G, distal (LTA-negative) tubules are designated d. The scale bar represents 100 microns.

**Figure 2:**
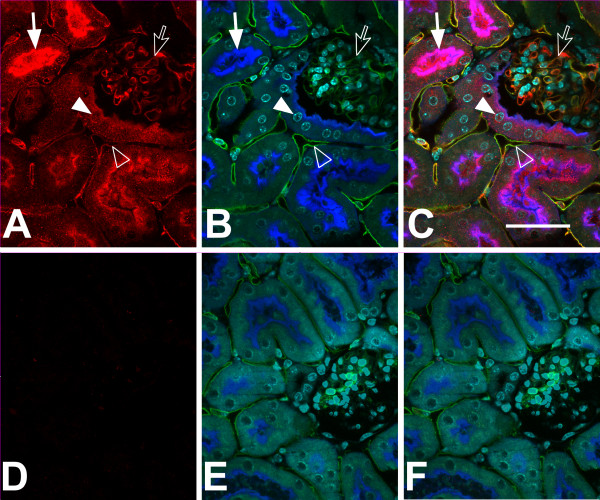
**Immunolocalization of CLIC4 in mouse kidney, high power images.** Vibratome sections stained with affinity-purified antibody to CLIC4 (red) plus markers for proximal tubule brush border (Lectin LTA, here shown in blue), endothelial cells (Lectin IB4, green), and nuclei (DAPI, cyan) were observed with a 60× objective under confocal microscopy. The upper set of images **(A-C)** are from a WT mouse, the lower set **(D-F)** are from a *Clic4* null mouse. **A** and **D** (left): CLIC4 signal alone (red); **B** and **E** (center): merged image of the three markers (proximal tubule brush border in blue, endothelial cells in green, and nuclei in cyan); **C** and **F** (right): merged image of all signals. Solid arrow: colocalization of CLIC4 with LTA in proximal tubule brush border. Solid arrow head: CLIC4 signal in nucleus of a proximal tubule cell. Open arrow: colocalization of CLIC4 and IB4 in glomerular capillary loop. Open arrowhead: colocalization of CLIC4 and IB4 in peritubular capillary. Scale bar represents 50 microns.

**Figure 3:**
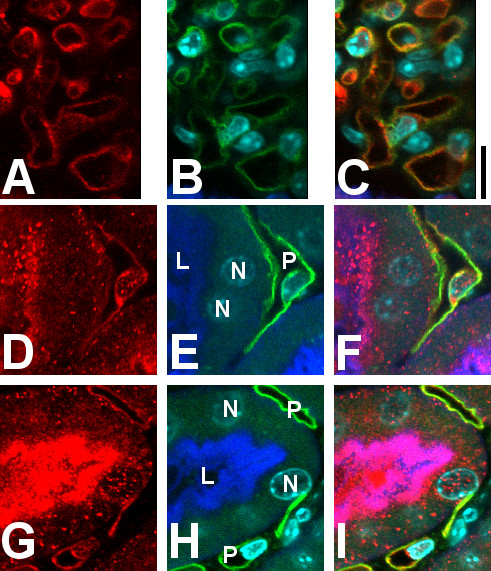
**Higher magnification images of CLIC4 in WT kidney.** Pseudocolor as in Figure 2. Upper set of images **(A-C)** shows glomerular capillary loops, lower sets **(D-F and G-I)** show a portion of a proximal tubule and underlying peritubular capillaries. Panels **A**, **D**, and **G** (left column): CLIC4 signal alone; Panels **B**, **E**, and **H** (center column): merged image of the three markers (proximal tubule brush border in blue, endothelial cells in green, and nuclei in cyan); Panels **C**, **F**, and **I** (right column): merged image of all signals. Structures are indicated in the center image: L indicates proximal tubule lumen, N indicates proximal tubule cell nuclei, P indicates peritubular capillary. Note the close colocalization of CLIC4 (red) with IB4 (green) in both glomerular capillary loops (upper image set) and peritubular capillary (lower image sets). Also, note presence of CLIC4 (red) colocalizing with the brush border (blue) and in the nuclei (cyan) of the proximal tubule cells and in the nuclei of the peritubular capillary endothelial cells in the two lower image sets. Scale bar to the right of the upper panel represents 10 microns.

Vibratome sections of kidney were prepared from 8 week old WT and *Clic4* null male mice and stained with CLIC4 antibody plus lectin markers of endothelial cells (IB4) and proximal tubule brush border (LTA), as well as a nuclear marker (DAPI). Images were collected with confocal microscopy and shown in Figures 1, 2 and 3. Identically treated, stained, and imaged sections from *Clic4* null mice served as the negative control and showed no significant signal with the CLIC4 antibody. Figure 1 shows low power images of the cortical labyrinth stained with antibodies to CLIC4 (red) plus the proximal tubule brush border (green) and nuclear (cyan) markers. Images from the wild type mouse are on the left, identically processed images from the *Clic4* null mouse on the right. Most of the tubules in the image are proximal tubules that are positive for the PTC brush border marker, LTA (green). A few LTA-negative distal nephron tubule cross sections are seen, labelled ‘d’ and glomeruli are labelled ‘G’. CLIC4 is detected in a subset of the LTA-positive proximal tubule segments where it shows an apical distribution. The tubule segments which express apical CLIC4 most prominently tend to be near glomeruli, suggesting they likely represent earlier segments of the proximal tubule. Whether the proximal tubule cells without prominent apical staining express CLIC4 in a diffuse cytoplasmic pattern is uncertain since the signal in not markedly more intense than the background signal in the *Clic4* null section. CLIC4 staining is also detectable in glomeruli and in an interstitial pattern consistent with the peritubular capillary network. CLIC4 signal is absent in the distal tubules. In the *Clic4* null mice, there is a low intensity diffuse signal in the proximal tubule cells, but the apical staining pattern and the glomerular and pertitubular staining patterns are absent.

Higher power images are presented in Figure 2, stained for CLIC4 (red), the proximal tubule brush border marker LTA (this time shown in blue), the endothelial marker IB4 (green) and the nuclear marker DAPI (cyan). Kidney from a wild type mouse is in the upper set of images, *Clic4* null in the lower set. In epithelial cells, CLIC4 (red) is prominent in the proximal tubules, identified by brush border labeling with the lectin LTA (blue). Within the proximal tubule cells, CLIC4 is strikingly apically polarized and appears to be present in the brush border where it colocalizes with the LTA marker (solid arrow). In addition, it is present in the cytoplasm in a punctate pattern consistent with a vesicular distribution. CLIC4 signal is also found in nuclei at about the same abundance as in the surrounding cytoplasm and many nuclei have punctate staining (solid arrow head) that is not present in the *Clic4* null controls. In addition to proximal tubule epithelium, CLIC4 signal is also abundant in endothelial cells of the peritubular capillary network (open arrowhead) and of the glomerular capillaries (open arrow) where it colocalizes with the endothelial marker IB4 (green). Each of these CLIC4 signals is absent in the identically processed images from the *Clic4* null kidney shown in the lower panel.

Higher magnification images are shown in Figure 3. In glomeruli (upper panel) the CLIC4 staining (red) colocalizes with the endothelial marker (green), and appears entirely confined within the capillary loops, indicating that the staining is truly in endothelial cells and not in podocytes or mesangial cells. The lower panels show two examples of a proximal tubule and neighboring peritubular capillary. Apical co-localization of the CLIC4 signal (red) with the brush border marker (blue) is again evident. Less intense punctate staining is present both in the cytoplasm and the nuclei of the proximal tubule cells at about the same intensity. Peritubular capillary endothelia stain throughout with the CLIC4 antibody (red), colocalizing with IB4 (green), and there is punctate staining of the endothelial cell nuclei.

### CLIC4 in growth and development

We had previously reported that *Clic4* null mice were underrepresented in the offspring from *Clic4* heterozygous parents, and that adult *Clic4* null male mice were smaller than littermate WT or *Clic4* heterozygotes [13]. The lower weight among *Clic4* null males was apparent by 5 weeks of age and persisted throughout life.

A cohort of age-matched 10–11 week old mice were used to assay whether the kidneys were smaller in the absence of CLIC4. Male and female mice of each genotype were studied as shown in Table 1. Both body and kidney mass of *Clic4* null mice were smaller than those of WT mice in both sexes. Kidney-to-body mass ratio was significantly lower in the male mice but not different in the female mice.

**Table 1:** Body and kidney mass of adult mice

	**WT**	**C4 null**	**P value**
**Males n**	10	9	
**Body mass (gm)**	45.6 ± 1.3	34.9 ± 1.3	0.00002
**Kidney mass (gm)**	0.776 ± 0.031	0.533 ± 0.019	0.000006
**Kidney/Body (%)**	1.70 ± 0.04	1.53 ± 0.03	0.0023
**Females n**	10	10	
**Body mass (gm)**	35.9 ± 1.3	27.8 ± 0.8	0.00004
**Kidney mass (gm)**	0.446 ± 0.016	0.360 ± 0.019	0.0032
**Kidney/Body (%)**	1.24 ± 0.03	1.30 ± 0.06	NS

### CLIC4 and renal angiogenesis

Absence of CLIC4 has been previously shown to impair angiogenesis; CLIC4 has been implicated in the intracellular tubulogenesis of endothelial cells [17] and is present in both glomerular and peritubular endothelial cells in the kidney (shown above). Thus, it is plausible that *Clic4* null mice may have impaired renal angiogenesis that could affect both kidney size and susceptibility to acute kidney injury. Decreased angiogenesis might be reflected in the total number of glomeruli and/or by decreased capillary density in the kidney.

**Figure 4:**
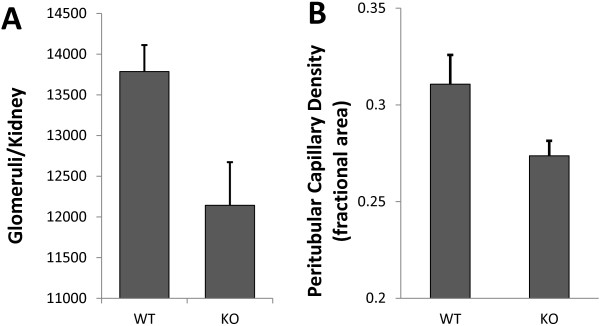
**Glomerular count and peritubular capillary density of WT and *Clic4 *null mice. A**. Glomeruli per kidney was determined by counting Alcian blue-stained glomeruli in aliquots of macerated kidney. WT: 13,785 ± 325 (n = 7) glomeruli per kidney; *Clic4* null: 12,142 ± 531 (n = 7) glomeruli per kidney (P = 0.022). **B**. Fractional area of a central longitudinal section of kidney stained with the endothelial cell marker CD31. WT: 31.1 ± 1.5% (n = 8); *Clic4* null: 27.4 ± 0.8% (n = 8) (P = 0.047).

Age-matched adult male WT and *Clic4* null mice (7 of each genotype) were used to determine glomerular counts. Glomeruli were stained by post-mortem perfusion of the mice with Alcian Blue followed by maceration of the kidneys in hydrochloric acid and then counting blue-stained glomeruli in aliquots of the resulting suspension. Results are shown in Figure 4. WT mice were found to average 13,785 ± 325 glomeruli per kidney while *Clic4* null mice had 12,142 ± 531 glomeruli per kidney (P = 0.022). Thus the *Clic4* null mice were found to have 12% fewer glomeruli than the WT mice.

**Figure 5:**
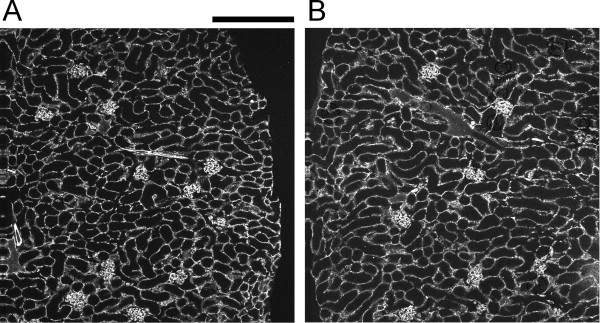
**Representative images of CD31-stained kidney sections used to generate the peritubular capillary density data.** The fraction of the kidney cross sectional area stained for CD31 was determined after excluding glomeruli and large vessel. **A**. Section from a WT kidney. **B**. Section from a *Clic4* null kidney. Scale bar represents 200 microns.

Peritubular capillary density was determined by quantitative image analysis of fluorescently stained kidney sections. Eight WT and *Clic4* null age-matched mice (4 male and 4 female of each genotype) were used in the experiment. Kidneys were harvested and longitudinal frozen sections through the center of each kidney were stained for CD31, a marker of endothelial cells. A series of contiguous images covering the entire length of the section from pole to pole was collected from each kidney. Representative images from the renal cortex are shown in Figure 5A and B. The fraction of the surface area of the kidney section stained with the endothelial cell marker, excluding glomeruli and large vessels, was determined. Results are shown in Figure 4B. 31.1 ± 1.5% of the wild type kidney sections and 27.4 ± 0.8% of the *Clic4* null kidney sections consists of capillaries (P = 0.047). Female mice tended to have a less dense peritubular capillary network than males, but this difference did not reach the 95% confidence level in either genotype.

### CLIC4 and proteinuria

**Figure 6:**
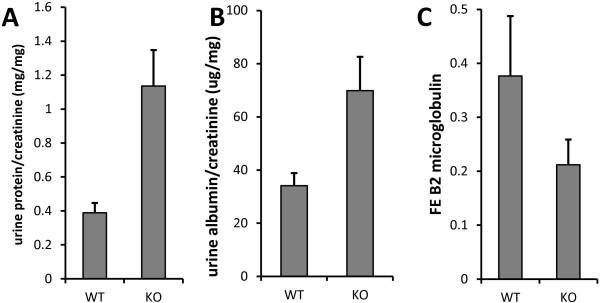
**Proteinuria in *Clic4 *null mice. A**. Total protein to creatinine ratio in urine from young adult male mice. WT: 0.296 ± 0.030 mg/mg (n = 17); *Clic4* null: 1.074 ± 0.182 mg/mg (n = 17), P = 0.00019. **B**. Urine albumin to creatinine ratio using a different set of male mice. WT: 34.1 ± 10.7 μg/mg (n = 5); *Clic4* null 69.8 ± 28.5 μg/mg (n = 5) (P = 0.030). **C**. Fractional excretion of β2 microglobulin in the same mice used in panel **B**. WT: 0.37% ± 0.11 (n = 5) *Clic4* null: 0.21% ± .04 (n = 5) (n.s.).

Mice were examined for the presence of proteinuria. Urine was collected from age-matched young adult male mice (6–10 weeks old) and the creatinine and protein concentrations determined. Results are presented in Figure 6A. Urine protein-to-creatinine ratios were 0.296 ± 0.030 mg/mg in WT (n = 17) and 1.074 ± 0.182 mg/mg in *Clic4* null (n = 17), P = 0.00019. Thus *Clic4* null mice have about 3.5 fold increased proteinuria compared to WT. To examine whether this represents glomerular or tubular proteinuria, the urine albumin-to-creatinine ratio and the fractional excretion of β2 microglobulin were determined among a different cohort of 5 age-matched male mice of each genotype (presented in Figure 6B and C). Urine albumin-to-creatinine ratios were 34.1 ± 4.8 μg/mg for the WT mice and 69.8 ± 12.8 μg/mg for the *Clic4* null mice (P = 0.030). Fractional excretions of β2 microglobulin were 0.37% ± 0.11 (n = 5) for the WT and 0.21% ± 0.04 (n = 5) for the *Clic4* null (n.s.). The albumin-to-creatinine ratio in the urine is significantly increased while fractional excretion of β2 microglobulin is not significantly different.

**Figure 7:**
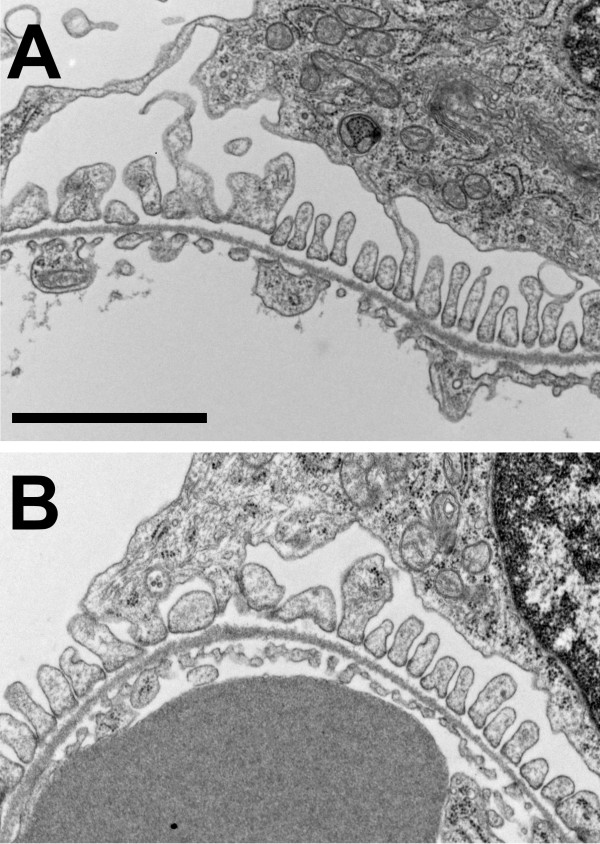
**Electron micrographs of glomeruli from WT and *Clic4 *null mice. A**. Wild type. **B**. *Clic4* null. In each, a section of a glomerular capillary is shown with the blood space below and the urinary space above, separated by basement membrane. The urinary surface of the basement membrane is lined with well-formed podocyte foot processes. The blood surface is lined with fenestrated endothelium. Scale bar represents 2 microns.

Ultrastructure of glomeruli from matched 6 week old WT and *Clic4* null mice was examined as shown in Figure 7. We could find no consistent differences between the WT and *Clic4* null glomeruli. In particular, both podocytes and glomerular endothelial cells were indistinguishable with neither prominent foot process effacement nor systematic changes in endothelial fenestrae.

### Acute kidney injury

A total of 46 *Clic4* null mice of 6.5 to 11.5 weeks of age (23 male, 23 female), and 46 age- and sex-matched WT mice were subjected to folic acid injury using intraperitoneal injections of 30 mg/ml folic acid dissolved in 300 mM sodium bicarbonate at a dose of 250 mg folic acid per kg body weight in two separate experiments. Blood samples were taken before the experiment and at 2, 7, and 21 days for blood urea nitrogen (BUN) determination. Mice were sacrificed at 21 days at which time kidneys were weighed and processed for histology. Baseline characteristics of the mice are shown in Table 2. Baseline BUN concentrations were no different between the WT and *Clic4* null mice and the two populations were well matched for sex, age, and weight.

**Table 2:** AKI following folic acid injection

	**WT (n = 46)**	**KO (n = 46)**	**P value**
**Baseline BUN**	26.0 ± 0.81	26.3 ± 1.13	NS
**Baseline age in weeks (mean, range)**	8.4, 7–11.5	8.3, 6–11	NS
**Baseline weight in grams (mean, range)**	29.8, 22.1–40.6	29.6, 22–39.5	NS
**Day 2 BUN**			0.021
**Mean**	109	187	
**Median**	65	143	
**No. BUN < 50**	20 (43%)	11 (24%)	0.077
**No. BUN > 200**	7 (15%)	20 (43%)	0.0055
**No. dead with renal failure within 7 days**	1 (2.2%)	7 (15%)	0.059

**Figure 8:**
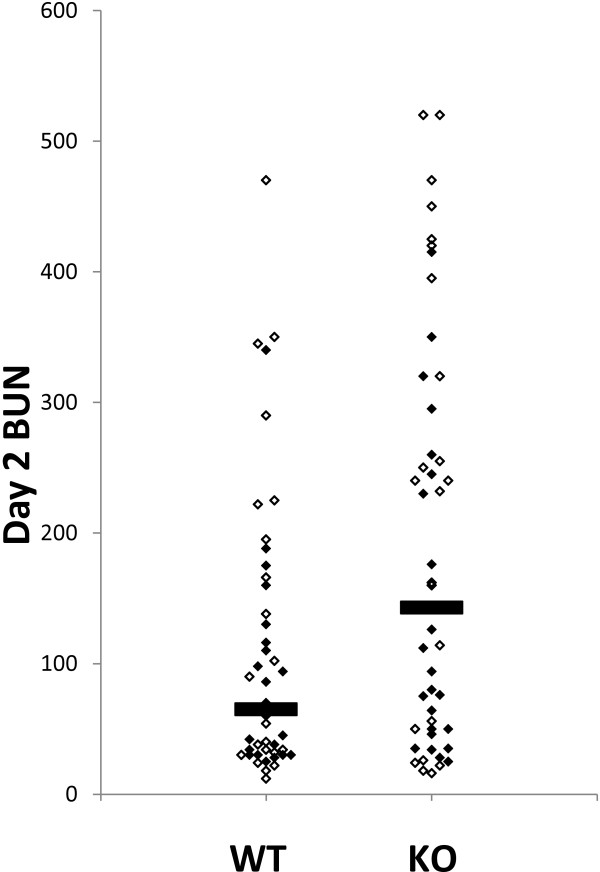
**BUN values at 48 hours after folic acid injection.** Each mouse is represented by a data point, females with the open symbols, males with the filled symbols. The horizontal bars mark the medians of each group. P = 0.021 by Wilcoxon rank sum test.

The day 2 BUN results are shown in Figure 8 and summarized in Table 2. There is a marked heterogeneity in the degree of acute kidney injury in response to intraperitoneal folic acid within each population. Fully 43% (20 out of 46) of the wild type mice and 24% (11 out of 46) of the *Clic4* null mice had minimal acute kidney injury with day two BUN less than 50 mg/dl (N.S., P = 0.077). Conversely, 13% of the WT mice and 43% of the *Clic4* null mice had severe kidney injury with the day two BUN values greater than 200 mg/dl (P = 0.0055). The mean 48 hour BUNs for the WT and *Clic4* null mice are 109 mg/dl and 187 mg/dl and the medians are 65 mg/dl and 143 mg/dl, respectively (P = 0.021 using Wilcoxon non-parametric testing). One WT mouse (2.2%) and 7 *Clic4* null mice (15.2%) died with severe AKI within one week of folic acid injection (P = 0.059). In sum, the *Clic4* null mice are significantly more susceptible to folic acid-induced acute kidney injury with higher average and median blood urea at 48 hours after injection, and a significantly higher fraction with severe injury. In addition the *Clic4* null mice show a trend toward fewer mice with minimal injury and more mice dying of acute injury within one week of folic acid injection, although these do not reach the 95% confidence level.

In both WT and *Clic4* null mice, the females tended to have more severe acute injury than the males as reflected in the day 2 BUN but this trend did not approach the 95% confidence level in either genotype using Wilcoxon non-parametric testing.

### Distribution of CLIC4 following tubular injury

**Figure 9:**
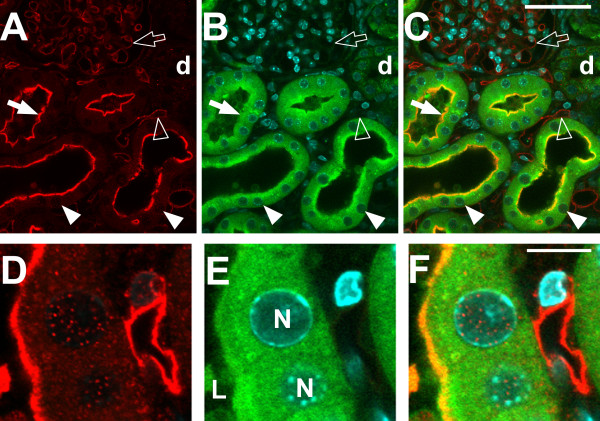
**Immunolocalization of CLIC4 in WT mice 48 hours after folic acid induced acute kidney injury.** Vibratome sections stained with affinity-purified antibody to CLIC4 (red) plus markers for proximal tubule brush border (Lectin LTA, blue) and nuclei (DAPI, cyan). A set of lower magnification image is shown in the upper set **(A-C)** and a higher magnification set from the same kidney in the lower set **(D-F)**. **A** and **D** (left column): CLIC4 signal (red) alone; **B** and **E** (center column): merged image of brush border (green) and nuclear (cyan) markers; **C** and **F** (right column): merged image of the CLIC4 signal with markers. In the upper set, solid arrow: CLIC4 is tightly polarized to the apical membrane of the dilated proximal tubule which has lost its brush border; solid arrowhead: nuclear localization of CLIC4 is less prominent than in uninjured kidney; open arrow: CLIC4 in a glomerular capillary loop; open arrowhead: CLIC4 in a peritubular capillary. In the lower set, L designates the lumen of the proximal tubule and N labels proximal tubule cell nuclei. A pertiubular capillary is present to the right.

Vibratome sections from wild type mouse kidney two days after folic acid exposure stained for CLIC4 (red), the proximal tubule marker LTA (green) and the nuclear marker DAPI (cyan) are shown in Figure 9. Lower magnification images are shown in the upper panel. The presence of significant tubular injury is reflected in these images as dilated tubules and loss of brush border. Other fields not presented here showed additional typical features of acute tubular injury including presence of sloughed cells in the tubule lumen and some areas where basement membrane appears to be devoid of epithelial cell covering. With loss of the brush border, LTA staining of is less prominent but enough residual staining remains to easily identify the proximal tubule as opposed to unstained distal tubules (indicated d). CLIC4 expression is still prominent in the injured proximal tubule segments and the apical polarization of distribution is even more marked, with the majority of CLIC4 appearing to reside in or immediately beneath the apical plasma membrane (solid arrow). In contrast to the uninjured kidney, CLIC4 is easily detected throughout the proximal tubule including the more distal straight segments (not demonstrated). Whether this represents up-regulation of CLIC4 in these cells, or more pronounced polarization of distribution is not clear. Contrary to predictions of our initial hypothesis, the modest nuclear staining in proximal tubule cells noted in the un-injured kidney (see Figures 2 and 3) has not dramatically changed, and is certainly not more prominent. Endothelial staining for CLIC4 in both peritubular (open arrowhead) and glomerular (open arrow) capillaries appears to be unchanged following injury. The lower panel shows higher magnification images including a segment of proximal tubule epithelium with underlying peritubular capillary, demonstrating persistent punctate nuclear staining which is not significantly different from that seen in uninjured kidney.

### Recovery from acute injury

**Figure 10:**
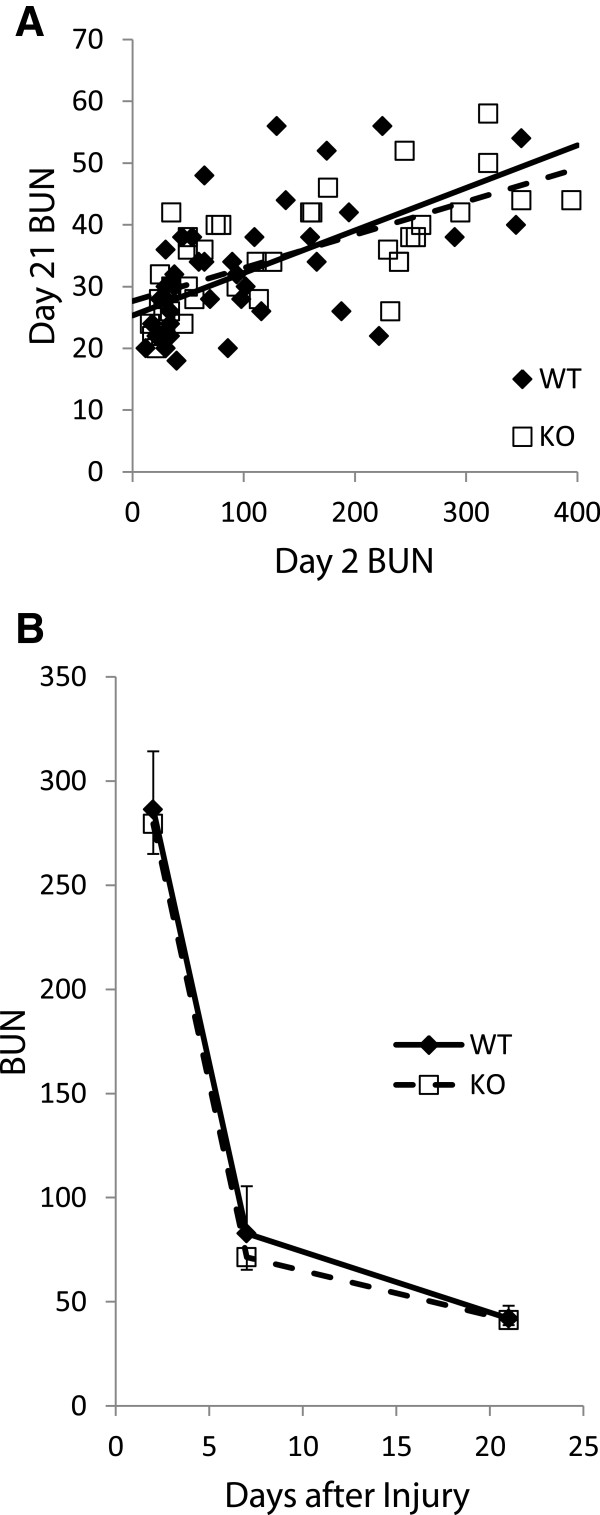
**Functional recovery following acute kidney injury. A**. The BUN values at day 21 are plotted as a function of the BUN at day 2 for each mouse. Solid line represents linear regression for data from WT mice; dashed line for *Clic4* null mice. The slopes of the lines are: WT 0.069 ± 0.015; KO 0.054 ± 0.009 (NS). **B**. Average BUN values for all mice with day 2 BUNs greater than 100 who survived to day 21. Standard errors are shown in only one direction to improve legibility. There is no significant difference between the average values at any time point.

Since the degree of injury is so heterogeneous, assessment of the recovery from injury is not straightforward. To analyze functional recovery, we first looked at the BUN at 21 days as a function of the BUN at day 2. Data for all the mice that survived to 21 days is plotted in Figure 10A. As expected, mice with greater initial injury had higher BUN at 21 days, although all the mice showed very significant functional recovery with none of the mice having a 21 day BUN greater than 60 mg/dl. The slopes of the linear regression lines derived from these data are 0.069 ± 0.015 for the WT and 0.054 ± 0.009 for the *Clic4* nulls, which are not significantly different, indicating that there is no significant difference between these populations in the extent of renal recovery as a function of the severity of initial injury.

To examine recovery in a different way, we limited our analysis to animals which suffered severe injury with a BUN of 200 mg/dl or greater on day 2 and who survived to day 21. Five WT mice and 13 *Clic4* null mice met these criteria. Average BUN values on days 2, 7, and 21 are plotted in Figure 10B. Both WT and *Clic4* null mice which survived severe initial injury showed good recovery of kidney function and the BUN values did not differ between the two groups at any time point. Thus extent and rate of functional recovery as reflected by BUN was not different between the WT and *Clic4* null mice.

**Figure 11:**
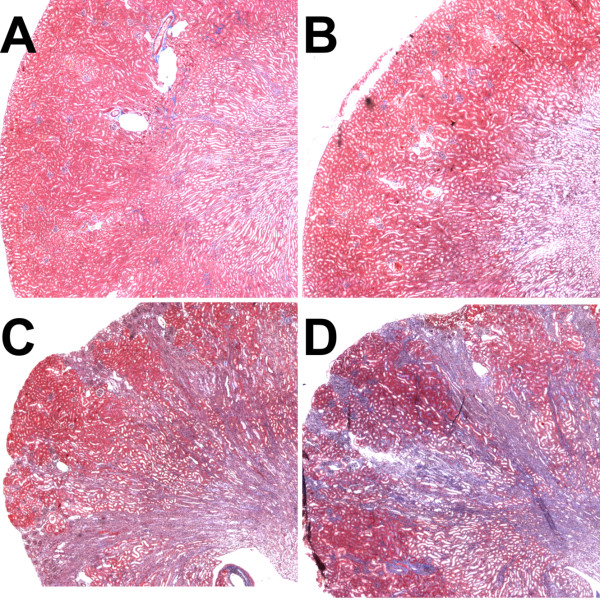
**Chronic scarring following acute kidney injury.** Central longitudinal kidney sections were prepared at 21 days after folic acid injury and stained with Mason’s Trichrome, which stains collagen blue. The upper panels show representative kidneys of mice sustaining minor injury and the lower panels are from mice sustaining severe injury; WT mice on the left, *Clic4* null mice on the right of each pair. **A**. WT mouse with day 2 BUN of 30 mg/dl, day 21 BUN 20 mg/dl, and section scored at 2.8% fibrosis. **B**. *Clic4* null mouse with day 2 BUN of 34 mg/dl, day 21 BUN 26 mg/dl, and section scored at 2.1% fibrosis. **C**. WT mouse with day 2 BUN of 350 mg/dl, day 21 BUN 54 mg/dl, and section scored at 38% fibrosis. **D**. *Clic4* null mouse with day 2 BUN of 395 mg/dl, day 21 BUN 44 mg/dl, and section scored at 32% fibrosis.

**Figure 12:**
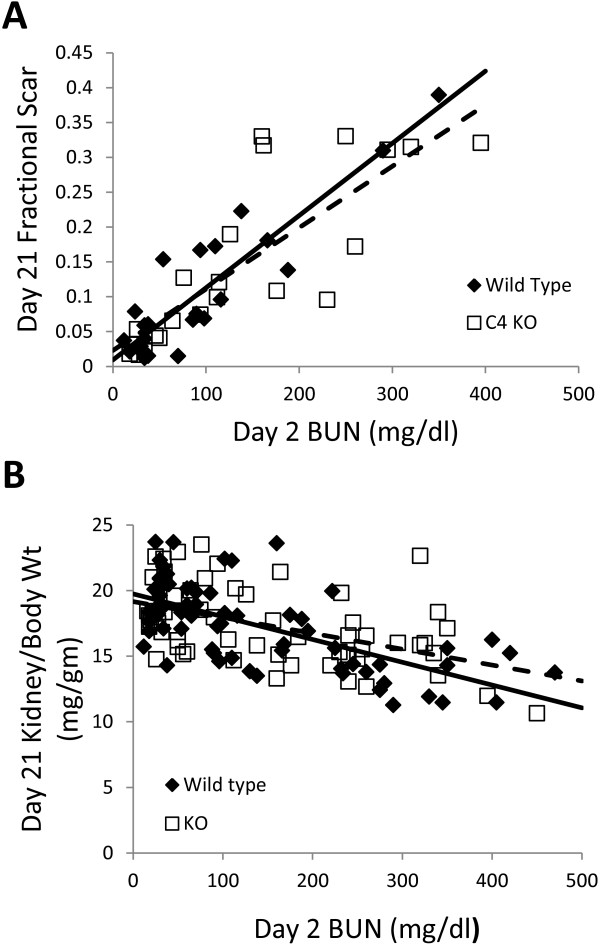
**Quantification of chronic scarring following acute injury. A**. Fractional area of a central longitudinal kidney section stained blue by Mason’s trichrome is plotted as a function of the day 2 BUN. **B**. Ratio of day 21 total kidney mass to pre-injury body mass is plotted as a function of the day 2 BUN for each mouse. In both panels A and B, WT mice are represented by filled diamonds, *Clic4* null mice by open squares. Solid line represents linear regression line for the WT, dashed line represents the linear regression line for the *Clic4* null. See text for values and standard error of the slopes. The slopes of the linear regression lines are not different between the WT and *Clic4* null mice in either graph.

At 21 days after injury, mice were euthanized and kidneys harvested. After weighing, kidneys were fixed. Longitudinal sections were obtained through the center of the kidney and stained with Mason’s Trichrome to assess extent of fibrosis. Typical interstitial fibrosis was found that correlated with the degree of initial injury – mice with minimal initial rise in BUN showed minimal scarring while mice with markedly elevate day 2 BUNs had more significant fibrotic scars. Representative images of WT and Clic4 null kidneys harvested 3 weeks after severe injury are shown in Figure 11. The fraction of the cross sectional area that was occupied by fibrosis was determined for a subset of the mice (23 WT and 22 CLIC4 nulls). The median fractional fibrosis was 6.85% and 10.37% for the WT and CLIC4 null mice, respectively, which did not reach statistical significance using Wilcoxon non-parametric testing. However, the marked difference in susceptibility to initial injury rendered this observation difficult to interpret. As with the functional recovery discussed above, we analyzed the degree of chronic fibrosis as a function of the initial injury to determine whether there may be a difference in fibrosis. Fractional fibrosis plotted against the day 2 BUN is shown in Figure 12A. As expected, there is a strong correlation of extent of fibrosis with the degree of initial injury. The slope of the regression lines are 0.00104 ± 0.0001 and 0.000801 ± 0.00014 for the WT and *Clic4* null mice, respectively, with the difference not approaching significance at the 95% confidence level.

Long-term renal scarring and fibrosis results in loss of renal mass, which is more easily and unambiguously quantified than are histologic scarring indices. To ask indirectly whether the absence of *Clic4* affects post-AKI scarring, we analyzed the relationship between the extent of initial injury and renal mass at 21 days after injury.

Our analysis was initially limited by the low number of WT mice that suffered severe injury. In order to increase the population with severe initial injury, we attempted to increase the intensity of the toxic exposure. In pilot experiments, we found that simply increasing the amount of folic acid solution at the same concentration had little effect on extent of kidney injury. Even doubling the dose did not appreciably change the fraction of mice suffering severe injury. In contrast, using the same dose of folic acid, but administering it in a more concentrated solution greatly increased toxicity.

We injected 29 WT mice (14 females, 15 males) and 31 *Clic4* null mice (15 females, 16 males) from the same population that was used for the large scale experiments described previously. The female mice received 250 mg/kg folic acid at 40 mg/ml in 300 mM sodium bicarbonate, and the male mice received 250 mg/kg folic acid at 50 mg/ml in 300 mM sodium bicarbonate. The baseline characteristics of these mice were as follows: WT average age 9.3 weeks (range 6–15.5), average weight 28.9 gm (range 21.1–39.9); *Clic4* null average age 10.0 weeks (range 7 – 15.1), average weight 30.7 (range 21.8–38.5). These mice suffered much more significant initial injury on average than the initial cohort given the same dose at 30 mg/ml. 56% of the wild type mice and 65% of the *Clic4* null mice had day 2 day BUN values greater than 100 mg/dl (NS). The differences do not reach the 95% confidence level although the mean and median day 2 BUNs showed a trend toward more severe injury among the *Clic4* nulls (WT and KO means 249 mg/dl and 271 mg/dl; medians 260 mg/dl and 325 mg/dl, respectively). Twenty-three of the 29 WT and 20 of the 32 *Clic4* null mice survived to 21 days at which time the mice were sacrificed and kidneys harvested.

Final (day 21) kidney weight was normalized to mouse body weight on day 0 and plotted as a function of the BUN on day 2 following folic acid injection. For this analysis only, data were pooled from the entire population treated with folic acid from both dosing protocols that survived to day 21 (67 WT and 58 KO mice). Results are shown in Figure 12B. There is a noticeable relationship between initial injury and the kidney weight following recovery with more severely injured kidneys undergoing more significant loss of mass. Linear regression lines from the data are shown and have slopes of -0.0174 ± 0.0024 and -0.0121 ± 0.0028 for the WT and *Clic4* null, respectively, with the difference not approaching significance at the 95% confidence level. Thus the degree of scarring as reflected in chronic loss of renal mass for a given amount of acute injury is not significantly different between the WT and *Clic4* null mice.

### Molecular markers of response to acute injury

To look more directly for an effect of CLIC4 on TGFβ signaling following acute kidney injury, we assessed phosphorylation of the SMAD pathway. One of the proximal steps in intracellular TGFβ signal transduction is the phosphorylation of SMADs 2 and 3. In keratinocytes, it has been demonstrated that CLIC4, through interactions with the protein Schnurri, potentiates TGFβ signaling by increasing the half-life of phosphorylated SMADs 2 and 3. If this also occurs during TGFβ signaling following acute kidney injury, we would expect to find lower levels of phosphorylated SMADs 2 and 3 in the injured *Clic4* null mice than in injured WT mice.

**Figure 13:**
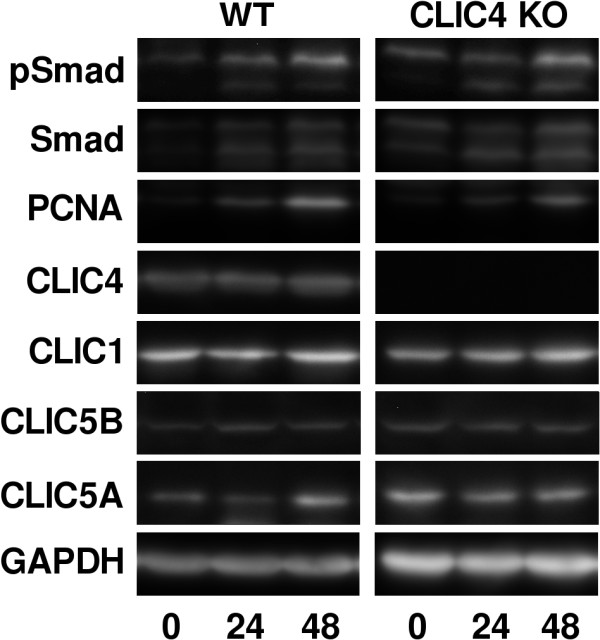
**Western blot images of SMADS, PCNA, and CLICs following acute kidney injury.** Representative results from a single WT and *Clic4* null mice from each time point are shown. The blots were sequentially stripped and probed with each antibody. In both the SMAD and PSMAD panels, the upper band represents SMAD2 and the lower band represents SMAD3.

**Figure 14:**
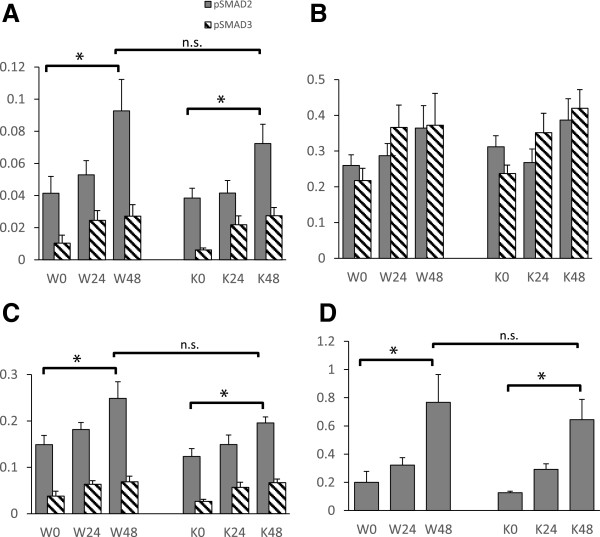
**Quantification of western blot signals for SMADs and PCNA.** Digitized chemiluminescent signals as in Figure 13 were normalized to the GAPDH signal from the same lane and plotted as arbitrary units. Values from wild type (W) and *Clic4* null (K) mice are shown at 0, 24, and 48 hours after folic acid injury as indicated. N = 8 for each group except for both 48 hour groups for which n = 7 (one mouse from each group was lost prior to analysis). **A**. pSMADs 2 and 3 normalized to GAPDH. **B**. Total SMADs 2 and 3 normalized to GAPDH. **C**. pSMADs 2 and 3 normalized to total SMADS 2 and 3. In panels A-C, the SMAD2 signal is shown with the solid bars, the SMAD3 signal with the hatched bars. **D**. PCNA. Error bars indicate SEM. Asterisk indicates comparison with P < 0.05. ns indicates not significant (P > 0.05).

A cohort of 48 age and sex-matched WT and *Clic4* null mice (12 males and 12 females in each group) were treated with the more toxic folic acid protocol noted above, (250 mg/kg folic acid at 50 mg/ml in 300 mM sodium bicarbonate given as an intraperitoneal injection) expected to cause severe injury in most mice. Baseline characteristics of the mice were as follows: WT mice, average age 8.4 weeks (range 7.6 – 9.7), average weight 32.4 gm (range 23.7 – 40.4); *Clic4* null mice, average age 8.6 weeks (range 6.1 – 10.1), average weight 31.8 (range 23.7 – 42.8). One third of the mice were sacrificed prior to injury, one third at 24 hours after injury, and one third at 48 hours after injury. Equal numbers of males and females were sacrificed at each time point. One female mouse of each genotype intended for the 48 hour time point died and was not included in the analysis. Kidneys were harvested and total protein prepared. Fifty micrograms of protein were separated by SDS-PAGE, blotted, and sequentially probed for total SMAD 2–3 (T-SMAD), phosphorylated SMAD 2–3 (P-SMAD), and GAPDH. Representative western blots are shown in Figure 13. In the T-SMAD and P-SMAD panels, SMAD 2 is the upper band and SMAD 3 is the lower band. The signals were normalized to the GAPDH signal as a loading control. Results for the entire data set are presented in Figure 14.

There was no significant difference in the level of total SMAD2 or 3 between the WT and *Clic4* null mice (Figure 14B). Total amount of both SMADs tended to increase in response to injury but this increase only reached the 95% confidence level at 48 hour time point for SMAD3 in the *Clic4* null mice. The levels of phosphorylated SMADs 2 and 3 normalized to GAPDH are shown in Figure 14A. The levels of both phosphorylated SMADs increased significantly over the 48 hours following injury. P-SMAD2 was more abundant than P-SMAD3 under all conditions. There appears to be a trend towards lower levels of P-SMAD2 in the *Clic4* null mice compared to wild type mice, but this difference did not approach the 95% confidence level at any time point.

To consider the data in a different way, the P-SMAD signals were normalized to the total SMAD (T-SMAD) signals and re-analyzed as shown in Figure 14C. The P-SMAD/T-SMAD ratio increased significantly by 48 hours after injury for SMAD2 and SMAD3 in both WT and *Clic4* null mice. There is a trend to lower P-SMAD2/T-SMAD2 ratio in the CLIC4 null mice compared to the WT mice, but this trend does not reach the 95% confidence level at any time point.

In addition to TGFβ signaling, CLIC proteins have been implicated in cellular proliferation, a process which also figures prominently in the response to acute kidney injury. To assess proliferation, we quantified expression of proliferating cell nuclear antigen (PCNA) in kidney homogenates, using western blotting as above. PCNA signals normalized to GAPDH are plotted in Figure 14D. PCNA significantly increases in kidney at 48 hours after injury but there is no significant difference in PCNA levels between WT and *Clic4* null mice.

### Expression of CLICs at baseline and following acute kidney injury

**Figure 15:**
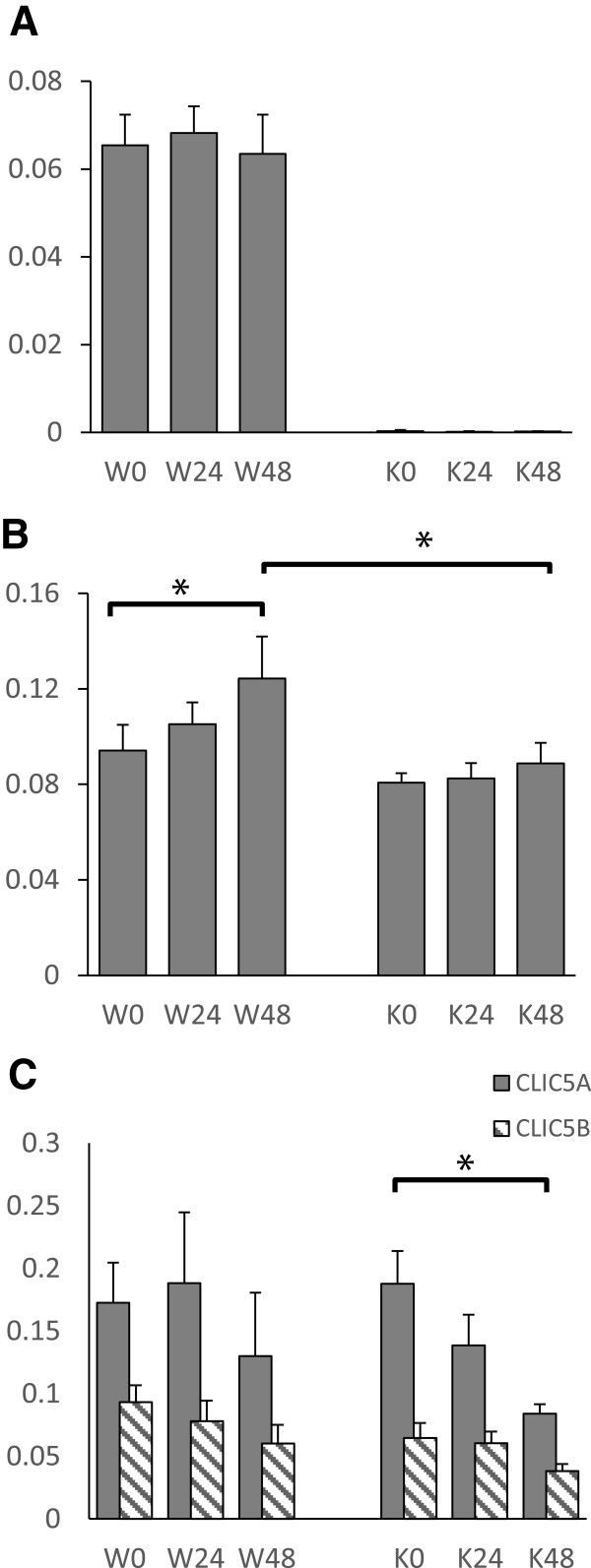
**Quantification of western blot signals for CLICs 1, 4, and 5.** Digitized chemiluminescent signals for the CLICs were normalized to the GAPDH signal in the same sample and plotted as arbitrary units. Values from wild type (W) and *Clic4* null (K) mice are shown at 0, 24, and 48 hours after folic acid injury as indicated. N = 8 for each group except for both 48 hour groups for which n = 7 (one mouse from each group was lost prior to analysis). **A**. CLIC4. **B**. CLIC1. **C**. CLIC5 **A** and **B**. CLIC5A is shown with the solid bars, CLIC5B with the hatched bars. Error bars indicate SEM. Asterisk indicates comparison with P < 0.05.

The CLIC family of proteins is very highly conserved (reviewed in [8]). It is possible that compensation between CLICs may account for some of the relative lack of effect of absence of CLIC4 on kidney function and response to injury. The same western blots used to probe for expression of SMADs and PCNA above were stripped and sequentially probed with antibodies to CLICs 1, 4, and 5 which are known to be expressed in the kidney [32,40-43]. The results are shown in Figure 15. As expected, CLIC4 (Figure 15A) is detected in the wild type mice and absent from the *Clic4* null mice at all time points. The level of expression of CLIC4 in the WT mice does not change in response to injury.

CLIC1 (Figure 15B) is present at comparable amounts in whole kidney lysates from uninjured WT and Clic4 null mice. Following injury of the WT mice, CLIC1 expression rises and is significantly higher at 48 hours than prior to injury. However, in the *Clic4* null mice, CLIC1 expression did not change significantly in response to injury and at 48 hours after injury, expression of CLIC1 is significantly higher in the WT than in the *Clic4* null mice.

CLIC5 is expressed in two different splice forms leading to two different proteins: a smaller gene product named CLIC5A, which very similar to CLIC1 and CLIC4, and larger gene product named CLIC5B containing an additional unique N-terminal region that includes an SH2 domain binding site that, when tyrosine phosphorylated, interacts with Src family kinases [44]. CLIC5A is known to be expressed in glomerular podocytes [42,43]. The distribution of expression of CLIC5B in kidney has not been reported. CLIC5A and CLIC5B (Figure 15C) are present in amounts that are not significantly different in whole kidney homogenates of WT and *Clic4* null mice at baseline. The levels of expression of both forms of CLIC5 do not change significantly in response to injury in the WT mice. However, in the *Clic4* null mice, the drop in expression of CLIC5A following injury is much more prominent and the decline in level by 48 hours does reach the 95% confidence level. There is no significant difference in the CLIC5A or CLIC5B signals between WT and *Clic4* null mice at any timepoint.

### Summary of AKI experiments

The acute kidney injury experiments yielded two salient results: *Clic4* null mice are more susceptible to folic acid induced acute kidney injury, and the absence of CLIC4 has no apparent impact on recovery from acute injury, either in function or in extent of scarring measured histologically or as reflected in kidney mass. Furthermore, we did not find any significant differences in SMAD phosphorylation or PCNA expression between WT and *Clic4* null mice in response to acute injury, and injury itself did not affect the steady state level of CLIC4 protein in WT mice. There is no over-expression of CLIC1 or CLIC5 at baseline or following injury that would suggest compensation for the absence of CLIC4.

## Discussion

The primary findings of this study are that the absence of CLIC4 results in smaller kidneys with fewer glomeruli and less dense peritubular capillary network, increased proteinuria that is primarily albumin with no increase in β-2 microglobulinuria, and increased susceptibility to the acute kidney injury induced by folic acid with no difference in the functional or histologic recovery from acute injury.

### CLIC4 and susceptibility to acute injury

*Clic4* null mice were found to have differences in kidney structure that could contribute to increased susceptibility to acute injury. *Clic4* null mice of both sexes have significantly smaller body mass and smaller kidneys than do WT mice. Furthermore, male *Clic4* nulls have lower kidney to body mass ratio than do matched WT males. Thus, small kidney size may contribute to sensitivity to acute injury, even though baseline kidney function as estimated by steady state BUN levels is equivalent.

Small kidneys may be small because they have fewer glomeruli and nephrons, and reduced nephron number has been previously implicated as a risk factor for acute kidney injury. The recognized role of CLIC4 in angiogenesis suggests a mechanism by which *Clic4* null mice may have fewer glomeruli. During development, glomerulogenesis is thought to require coordinated interaction between the renal corpuscle developing from the epithelial compartment, and invading endothelial cells providing the vascular components. Failure or delay in endothelial invasion of the renal corpuscle could decrease the number of fully developed glomeruli. With this in mind, we determined the number of glomeruli in WT and matched *Clic4* null mice and found that the absence of CLIC4 is associated with a 12% decline in glomerular number in adults.

Impaired angiogenesis during development might also result in a less dense peritubular capillary network which may be a risk factor for susceptibility to acute kidney injury, and indeed we found that the absence of CLIC4 is associated with a 12% decrease in the fraction of longitudinal kidney sections that are occupied by peritubular capillaries. Absence of CLIC4 could potentially also effect the active angiogenic response to acute kidney injury [45]. Increased angiogenesis in the peritubular capillaries following acute folic acid nephrotoxicity in mice has been reported. This angiogenesis may be at least partially driven by changes in levels of angiopoeitin 1, vascular-endothelial growth factor A, and hypoxia inducible factor 1α which occur in the same time frame [6,45]. Whether this response has an impact on the severity of the acute injury itself or only on the chronic consequences of acute injury is uncertain.

### CLIC4 and proteinuria

Proteinuria has clearly been associated with increased risk of acute kidney injury both in human studies and in animal models [46]. We found that the urine protein-to-creatinine ratio of *Clic4* null mice was elevated more than 3 fold. Since CLIC4 is prominently expressed in both glomeruli and proximal tubules, it is conceivable that absence of CLIC4 could result in proteinuria induced by either glomerular dysfunction, tubular dysfunction, or both. To differentiate these two, we separately assessed albuminuria and β2 microglobulinuria. One would expect urinary albumin to be increased during proteinuria of either glomerular or tubular origin, while β2 microglobulinuria would only be increased during tubular proteinuria. We found that albuminuria as reflected by the urine albumin to creatinine ratio was significantly elevated among the *Clic4* null mice, while the fractional excretion of β2 microglobulin was unchanged. We conclude that *Clic4* null mice have proteinuria of glomerular origin, presumably a result of alterations in the glomerular capillaries as a consequence of the absence of CLIC4 from these cells. However, ultrastructural analysis failed to demonstrate the typical morphologic alterations in the structure of either the glomerular endothelial cells or podocytes that could explain the proteinuria.

There are some limitations to the conclusions regarding proteinuria that should be noted. First, the estimations of proteinuria in this study were entirely based on the ratio of protein to creatinine in the urine, not 24 hour urine collections. The inherent assumption is that steady state rates of creatinine production are similar between WT and *Clic4* null mice, an assumption that was not tested. Second, rather than increased glomerular albumin leakage, an alternative explanation for selective albuminuria without β2 microglobulinuria could be a selective defect in proximal tubule endocytosis that effects only the albumin endocytic pathway but the the β2 microglobulin pathway. Finally, low glomerular number itself has been associated with albuminuria in mice although a causal relationship is uncertain [47]. Thus it is possible that the modest proteinuria seen in the Clic4 null mice could be a consequence of the low glomerular number resulting from the absence of CLIC4 during development, and not an independent effect of absence of CLIC4 in the adult kidney.

### Folic acid model of acute kidney injury

We chose the folic acid model of acute injury because it uses a relatively non-toxic agent, is easy to administer to a large number of animals, and has been used with success in prior studies of acute kidney injury and subsequent fibrosis [37]. However, we found this model to possess some significant shortcomings. The marked variability in the extent of kidney injury to a fixed dose of folic acid rendered the data difficult to interpret. The degree of acute kidney injury as reflected by BUNs does not follow a Gaussian distribution. None-the-less, non-parametric statistical methods demonstrated a significant difference in the severity of acute injury as reflected in the 48 hour BUN values. Additional criteria suggest that the severity of injury is different between the two populations: the fraction of mice suffering severe acute injury is significantly different, and there are trends that do not quite reach the 95% confidence level that the fraction of mice suffering minimal injury is lower, and the fraction of mice dying with severe AKI within 7 days of injury are higher in the *Clic4* null population than in the WT. Therefore, the observation that *Clic4* null mice are more susceptible to folic acid-induced acute injury is strongly supported by the data. Factors contributing to the enhanced susceptibility to AKI are uncertain, but low glomerular/nephron number, low peritubular capillary density, and proteinuria have all been shown or suggested to increase risk of AKI in the past [46,48,49].

### CLIC4 and TGFβ signalling following acute kidney injury

The differences in initial injury between the populations, complicated by the marked variability of extent of injury within each population, made it very difficult to compare recovery and fibrosis between the WT and *Clic4* null populations. We used two methods of analysis to assess functional recovery while compensating for degree of initial injury. First, we looked at the extent of long term functional recovery as a function of initial injury and found no difference in this relationship between the WT and *Clic4* null mice. Second, limiting the analysis to those mice which suffered severe initial injury with day 2 BUNs greater than 200, we found no difference in the rate or extent of recovery of kidney function between WT and *Clic4* null mice.

Despite good functional recovery, histologic examination of kidneys 21 days after injury revealed extensive interstitial fibrosis in those mice that suffered severe initial injury. The fraction of the area of a longitudinal section that consisted of scar was determined. As expected, the extent of scarring correlated strongly with the degree of initial injury. However, there was no difference in the extent of chronic scarring as a function of the severity of the acute injury between the WT and *Clic4* null mice. Furthermore, using a larger population of mice with an increased number suffering severe injury, there no difference in the 21 day kidney-to-body-weight ratio (a surrogate marker of extent of scarring) as a function of severity of initial injury between the WT and *Clic4* nulls. Molecular analysis of the TGFβ signaling pathway failed to demonstrate a statistically significant difference in phosphorylation of SMADs 2 or 3 between WT and *Clic4* null mice following injury, and immunolocalization of CLIC4 in injured kidney tubules failed to show nuclear redistribution of the protein. Taken together, the data do not support a model similar to that of the keratinocytes in which a substantial fraction of CLIC4 is targeted to the nucleus where it significantly potentiates TGFβ signaling. Clearly the mice do not manifest the dramatic difference in scarring and fibrosis one might expect if CLIC4 plays a decisive role in potentiating TGFβ signaling in proximal tubule cells analogous to the data regarding cells of the skin. The absence of an important role for CLIC4 suggests tissue and cell specific patterns of TGFβ signaling where CLIC4 plays a role in some cell types but not others. Whether CLIC4 plays a meaningful role in this pathway in kidney cells *in vivo* in other experimental models remains to be determined, but our data indicate it does not have a major impact on the recovery from folic acid induced acute renal failure.

### Changes in expression of CLICs in response to injury in the presence and absence of CLIC4

We examined the levels of CLICs 1, 4, and 5 in whole kidney homogenates in response to acute folic acid injury in WT and *Clic4* null mice. Acute injury did not change level of expression of CLIC4 protein itself during the 48 hours following injury in the WT mice. We did not detect significant up-regulation of CLIC1 or CLIC5A/B in the absence of CLIC4 at baseline, indicating there is not a compensatory up-regulation of these CLICs in the absence of CLIC4 in uninjured kidney, at least at the level of steady state protein in the whole organ. However, we did see intriguing differences in response to injury for both CLICs 1 and 5 in the presence and absence of CLIC4. Expression of CLIC1 is substantially increased over the 48 hours following injury in WT mice, but this up-regulation is greatly impaired in the absence of CLIC4. Expression of both splice variants of CLIC5 are stable following injury in WT mice, but in the absence of CLIC4, there is a significant decrease in expression of CLIC5A and noticeable trend to decreased expression of CLIC5B. These data suggest presence of CLIC4 is permissive for up-regulation of CLIC1 and sustained expression of CLIC5 following acute injury. Since these data are from whole kidney lysates, we cannot know which cell types are responsible for these changes of expression.

## Conclusion

We have shown that *Clic4* null mice have increased susceptibility to acute kidney injury induced by folic acid. We found a number of differences in the *Clic4* null mice that would be expected to contribute to this increased susceptibility, including small kidneys, fewer glomeruli, a less dense peritubular capillary network, and proteinuria that appears to be primarily of glomerular origin. While we have found some differences in the *Clic4* null mice that could plausibly contribute to increased susceptibility to acute kidney injury, the response to acute kidney injury is complex and systemic, and CLIC4 is expressed in many tissues and cell types. Certainly it is possible that other, as of yet unrecognized direct or indirect consequences of the absence of CLIC4, both in renal and in extrarenal tissues, may have a decisive impact on these observations

Our original hypothesis that CLIC4 contributes substantially to fibrosis and long-term kidney scarring following injury is not persuasively supported by our data. We did not find the obvious difference in scarring one would expect if CLIC4 is a major, non-redundant determinant of the intensity and duration of TGFβ signaling in kidney cells. Furthermore, we did not find nuclear redistribution of CLIC4 in proximal tubule or endothelial cells following injury in the WT mice, and we did not find a significant difference in levels of pSMAD 2 or 3 at 24 or 48 hours following injury between WT and *Clic4* null mice. These data strongly challenge the hypothesis that CLIC4 potentiates TGFβ signaling in the kidney following acute injury.

## Abbreviations

TGFβ: Transforming growth factor β; DAPI: 4′,6-diamidino-2-phenylindole; BUN: Blood urea nitrogen; AKI: Acute kidney injury; PCNA: Proliferating cell nuclear antigen.

## Competing interests

The authors declare that they have no competing interests.

## Authors’ contributions

JCE served as the principal investigator and was directly involved in all phases of the work. JB processed the biochemical samples, carried out the western blotting and performed data analysis. PK managed the animals and performed the immunostaining and confocal microscopy. Y-W C also managed the animals, performed the glomerular counting and capillary density assessment. All authors contributed to experimental plan and interpretation and have read and approved the manuscript.

## Pre-publication history

The pre-publication history for this paper can be accessed here:

http://www.biomedcentral.com/1471-2369/15/54/prepub
